# The Missense Alteration A5T of the Thyroid Peroxidase Gene is Pathogenic and Associated with Mild Congenital Hypothyroidism

**DOI:** 10.4274/jcrpe.2017

**Published:** 2015-08-31

**Authors:** Hakan Cangül, Korcan Demir, H. Ömür Babayiğit, Ayhan Abacı, Ece Böber

**Affiliations:** 1 Medipol University Faculty of Medicine, Department of Medical Genetics, İstanbul, Turkey; 2 Dokuz Eylül University Faculty of Medicine, Department of Pediatric Endocrinology, İzmir, Turkey; 3 Dokuz Eylül University Faculty of Medicine, Department of Pediatrics, İzmir, Turkey

**Keywords:** congenital hypothyroidism, thyroid dyshormonogenesis, thyroid peroxidase

## Abstract

Congenital hypothyroidism (CH) occurs with a prevalence of approximately 1:4000 live births. Defects of thyroid hormone synthesis account for 15-20% of these cases. Thyroid peroxidase (TPO) gene is the most common cause for dyshormonogenesis. So far, more than 60 mutations in the TPO gene have been described, resulting in a variable decrease in TPO bioactivity. We present an 8-day-old male with mild CH who was identified to have a G to A transition in the fifth codon of the TPO gene (c.13G>A; p.Ala5Thr). The unaffected family members were heterozygous carriers of the mutation, whereas 400 healthy individuals of the same ethnic background did not have the mutation. Mutation analysis of 11 known causative CH genes and 4 of our own strong candidate genes with next-generation sequencing revealed no mutations in the patient nor in any other family members. The results of in silico functional analyses indicated partial loss-of-function (LOF) in the resulting enzyme molecule due to mutation. The patient’s clinical finding s were consistent with the effect of this partial LOF of the mutation. In conclusion, we strongly believe that A5T alteration in the TPO gene is actually pathogenic and suggest that it should be classified as a mutation.

## INTRODUCTION

Congenital hypothyroidism (CH) is the most common endocrine disorder of infancy and one of the most common preventable causes of mental retardation with a prevalence of 1:3000-1:4000 live births (1). 80% to 85% of CH cases are associated with disorders of thyroid gland development (dysgenesis) and the remaining 15-20% of cases are caused by defects in any step of thyroid hormone synthesis (dyshormonogenesis) (1,2). Occasionally, hypothalamic or pituitary disease leading to a deficiency of thyrotropin-releasing hormone or thyrotropin (TSH) might cause central (secondary) hypothyroidism (3).

Although most cases of CH are sporadic, dyshormonogenetic cases are often recessively inherited (3). Thyroid dyshormonogenesis has been linked to mutations in the sodium iodide symporter (NIS), SLC26A4 (which encodes pendrin, a multifunctional anion exchanger), thyroid peroxidase (TPO), dual oxidase 2 (DUOX2), DUOX maturation factor 1 (DUOXA1), DUOX maturation factor 2 (DUOXA2), dehalogenase 1 (DEHAL1) and thyroglobulin (TG) genes (4). Mutations in TPO appear to be the most common cause of dyshormonogenesis with permanent CH (5,6,7). More than 60 mutations have been described in the human TPO gene (1,4).

The missense alteration A5T is listed in the dbSNP database (rs369441749) and no phenotype information is currently available. Here, we discuss in detail a previously reported case of a patient with CH (4) who is carrying the A5T alteration homozygously and present several lines of evidence for the pathogenicity of this change in the TPO gene.

## CASE REPORT

This patient was enrolled through our studies among the list of genetics of CH (4,8,9,10,11,12,13,14,15,16,17,18,19,20).

This male patient had presented at age 8 days to our outpatient clinic with findings of an elevated serum TSH concentration of 80 mIU/L (normal <15 mIU/L) and a borderline serum free thyroxine (T4) (0.9 μg/dL, normal 0.9-2.6 μg/dL). He was the second-born (C-section, birth weight 3660 g) of healthy and first-degree consanguineous Turkish parents following an uncomplicated pregnancy. The 21-year-old G2P2 mother had no history of thyroid disease. The parents and the 3.5-year-old male sibling were reported to be in good health.

Physical examination revealed a weight of 3850 g (50th-75th percentile). Length was 58 cm (50th percentile). Anterior fontanel size was normal. There was no jaundice, umbilical hernia or macroglossia. Neonatal reflexes were normal. He had no goiter. The male genitalia appeared normal with descended testes. An ultrasound of the thyroid gland revealed a normally located gland of normal size located in the anterior neck. Thyroid scintigraphy with technetium pertechnetate was also normal.

Thyroid hormone replacement therapy was initiated with L-T4 (50 μg/day) and the dose was adjusted for age and weight during follow-up. The patient’s growth and psychomotor development proceeded normally. At his most recent visit at the age of 6 years, his height was 121 cm (75th-90th percentile) and his weight was 22.4 kg (50th-75th percentile). He was euthyroid while using 1.3 μg/kg/d L-thyroxine.

### Genetic Analyses

#### Potential Linkage Analysis

First we performed linkage analysis to all 11 known genetic loci for CH in all family members using microsatellite markers. Four primer pairs surrounding each locus were selected (Table 1). Fluorescent labelling of one oligonucleotide of each primer pair enabled the sizing of polymerase chain reaction (PCR) products in a capillary electrophoresis machine by the use of GeneMapper v4.0 software suite (Applied Biosystems, Warrington, UK). By combining genotypes for each microsatellite marker, we constructed haplotype tables for each family member. According to these tables and assuming autosomal recessive inheritance in consanguineous families, the family showed potential linkage to the TPO locus, i.e. the case was homozygous for all markers in this locus, while both parents and an unaffected brother were heterozygous carriers of same markers.

#### Direct Sequence Analysis of the Thyroid Peroxidase Gene

The DNA template of the TPO gene was downloaded from the Ensembl database (ENSG00000115705). All alternative transcripts (17 in total) were included to ensure that primers were designed to cover all coding exons and intron/exon boundaries. Intronic primers flanking the coding sequence were designed for PCR amplification using Exon Primer and Primer 3. Primer sequences and PCR conditions are available upon request. PCR products were size-checked on 1% horizontal agarose gels and cleaned up using MicroCLEAN (Microzone, Haywards Heath, UK) or gel-extracted using QIAquickTM Gel Extraction kit (Qiagen, Crawley, UK). The purified PCR products were sequenced in both forward and reverse directions using the ABI BigDye Terminator v3.1 Cycle Sequencing kits on an ABI Prism 3730 DNA Analyzer (Applied Biosystems, Warrington, UK).

The sequences were downloaded using Chromas software and the analysis showed a G to A transition in the fifth codon of the TPO gene (c.13G>A; p.Ala5Thr). The case was homozygous for this alteration, while both parents and the unaffected sibling were all heterozygous carriers as predicted by the linkage analysis. These results indicated the proper segregation of the mutation with the disease status in the family according to autosomal recessive inheritance pattern as expected in consanguineous families. The mutation was not present in 400 ethnically-matched control chromosomes. Neither the case nor the other family members carried any other mutation in the TPO gene.

#### Next Generation Sequencing for All Other Known Causative Congenital Hypothyroidism Genes

With the availability of next generation sequencing, we developed a comprehensive next generation sequencing-based strategy for genetic diagnosis in CH as described elsewhere (15). This test included full sequencing of all 11 known causative CH genes and 4 of our own strong candidate genes. Mutation analysis of these genes revealed no mutations in any of these genes neither in the patient nor in any other family members. These results implicated A5T as the only candidate alteration to cause the disease in the family.

#### In Silico Functional Analysis of the Mutation A5T

To examine the pathogenicity of the A5T change, we performed in silico functional analyses and predicted the effects on the structure of the TPO molecule. TPO is a member of the animal heme peroxidase family, which is expressed in the thyroid and involved in the processing of iodine and iodine compounds. There is no molecular structure of any TPO deposited in public databases to date. However, there are structures solved for close proteins contained in the same XPO peroxidase subfamily, namely myeloperoxidases and lactoperoxidases.

We used SDM and MOSST tools for functional analyses. The template experimental models were used for comparison to take into account the influence of ligands in the predictions, especially water molecules. In silico analysis was performed by replacing the alanine amino acid one by one and performing a rotamer search to select the more adequate rotamer for each substitution. For building the mutated protein structures, the program Andante was used. The pairs of wild-type mutant models were submitted to SDM independently. MOSST requires a multiple alignment of sequences of homologue proteins to perform the analysis. For this object, the 200 most similar homologue protein sequences detected by the BLAST search were used as input for the ClustalW algorithm to obtain a multiple alignment. This multiple alignment was re-formatted and submitted to MOSST for analysis. These 200 proteins included all members of the XPO subfamily of peroxidases described above, namely peroxidases, myeloperoxidases, eosinophil peroxidases and lactoperoxidases.

For amino acid position 5, MOSST predicts the highest probability for the presence of an alanine (with a 36.1% probability), which is the residue present in the wild-type consensus TPO sequence. All public databases currently report no minor allele frequency for this position. To confirm the predictions of MOSST, an analysis was performed to predict the presence of a functional signal peptide in the A5T TPO mutant protein sequence, using the algorithms SignalP-4.0, PrediSi, Phobius and Predotar. All of these algorithms confirmed both the presence of a signal peptide for the wild-type enzyme and partial disruption of this localization signal by A5T mutation. These data strongly suggest that A5T mutation causes partial loss-of-function (LOF) in the resulting TPO enzyme.

## DISCUSSION

The TPO gene (OMIM access number *606765) is located on the chromosome 2p25. It comprises 17 exons, covers approximately 150 kb of genomic DNA and codes 933 amino acids (21). The enzyme catalyses the oxidation, organification and coupling reactions. It is a glycosylated membrane-bound hemoprotein localized on the apical membrane of the thyrocyte (3). Mutations have been classified as missense, nonsense, frameshift and splicing mutations and gene deletions (5,21,22). So far, more than 60 different mutations of the TPO gene have been reported, for the most part occurring in exons 8, 9, 11 and 14 (22). The clinical spectrum ranges from mild to severe hypothyroidism depending on the severity of the mutation.

A5T change in the TPO gene is currently listed in the dbSNP database with the number rs369441749 and basic prediction tools such as PolyPhen and SIFT classify it as possibly benign. Although previously we reported A5T alteration as a benign variant, in this study, we present several lines of evidence for the pathogenicity of this alteration: (i) homozygosity of the mutation in the patient; (ii) proper segregation of both the mutation and the microsatellite marker haplotypes with the disease status in the family according to autosomal recessive inheritance pattern as expected in consanguineous families, i.e. the case was homozygous and all unaffected members of the family were heterozygous; (iii) absence of any other mutation of the TPO gene in the patient; (iv) absence of any mutation in all other known causative CH genes; (v) absence of the mutation in 400 ethnically-matched control chromosomes; (vi) the results of in silico functional analyses indicating partial LOF in the resulting enzyme molecule due to mutation; (vi) mild phenotype observed in the patient which is consistent with the effect of this partial LOF of the mutation.

Considering the above findings, we strongly believe that A5T alteration in the TPO gene is actually pathogenic and suggest that it should be classified as a mutation. Moreover, the phenotype of our patient implies that this mutation is associated with mild CH. Further reports of CH patients carrying the same mutation will help to establish a firmer genotype/phenotype relationship associated with this mutation.

## Figures and Tables

**Table 1:**
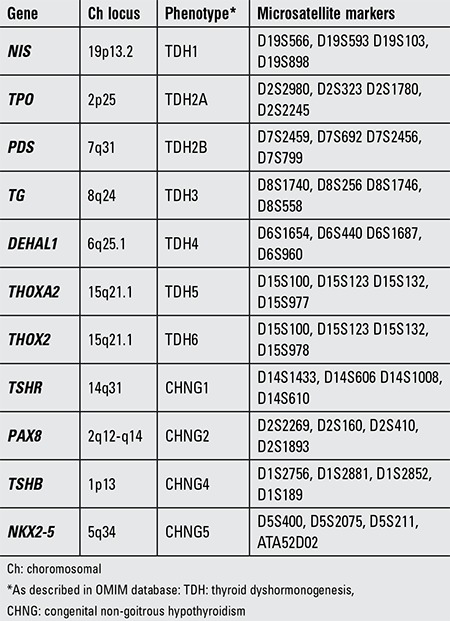
Genes causing congenital hypothyroidism, associated phenotypes and microsatellite markers used for their linkage analysis
